# Muscle strength, aerobic capacity, and exercise tolerance are impaired in left ventricular assist devices recipients: A pilot study

**DOI:** 10.3389/fphys.2022.967817

**Published:** 2022-08-08

**Authors:** Stefano Gobbo, Francesco Favro, Valentina Bullo, Lucia Cugusi, Andrea Di Blasio, Alessandro Bortoletto, Danilo Sales Bocalini, Andrea Gasperetti, Andrea Ermolao, Marco Bergamin

**Affiliations:** ^1^ Department of Medicine, University of Padova, Padova, Italy; ^2^ Department of Biomedical Sciences, University of Sassari, Sassari, Italy; ^3^ Department of Medicine and Sciences of Aging, G. D’Annunzio University of Chieti-Pescara, Chieti, Pescara, Italy; ^4^ Laboratorio de Fisiologia e Bioquimica Experimental, Centro de Educacao Fisica e Deportos, Universidade Federal do Espirito Santo (UFES), Vitoria, Brazil; ^5^ Sports and Exercise Medicine Division, Department of Medicine, University of Padova, Padova, Italy

**Keywords:** left ventricular assist device, exercise test, muscle strength, exercise tolerance, aerobic capacity, postural control

## Abstract

**Background:** Left ventricular assist devices (LVAD) are increasingly being used as a therapy for advanced heart failure, both as a bridge to heart transplant and, given the rapid advances in the LVAD’s functionality and safety, and constant lack in availability of donor organs, as long-term destination therapy. With the diffusion of such therapy, it is crucial to assess patients’ muscle strength, aerobic capacity and exercise tolerance, to improve their functional capacity.

**Methods:** 38 LVAD recipients (33 men and five women) were included. Exercise testing including a maximal cardiopulmonary exercise test (CPET), handgrip, isometric and isokinetic strength testing of knee and ankle flexion/extension, and Romberg balance test in three conditions (eyes open, eyes closed, double task). Given the small and heterogeneous final sample size, a mostly descriptive statistical approach was chosen.

**Results:** 12 participants were classified as “Obese” (BMI>29.9). The most common comorbidities were type II diabetes and chronic kidney disease. Only 12 participants were able to successfully complete all the assessments. CPET and isokinetic strength trials were the least tolerated tests, and the handgrip test the best tolerated. Mean VO_2_ peak was 12.38 ± 3.43 ml/kg/min, with 15 participants below 50% of predicted VO_2_ max, of which 6 below 30% VO_2_max. Mean handgrip strength was 30.05 ± 10.61 Kg; 25 participants were below the 25° percentile of their population’s normative reference values for handgrip strength, 10 of which were below the 5° percentile. Issues with the management of the external pack of the LVAD and its influence on the test limited the validity of the balance tests data, therefore, no solid conclusions could be drawn from them. VO_2_ peak did not correlate with handgrip strength or with any of the lower limb strength measures.

**Conclusion:** LVAD recipients show greatly reduced functional capacity and tolerance to exercise and exercise testing, with low overall strength levels. As strength variables appear to be independent from VO_2_ peak, different lower limbs strength tests should be explored to find a tolerable alternative in this population, which is subjected to muscle wasting due to old age, reduced tissue perfusion, side effects from the pharmacological therapies, and prolonged periods of bedrest.

## Introduction

Heart failure is a significant cause of morbidity and mortality, affecting at least 64 million patients in the world (Lippi and Sanchis-Gomar, 2020). Left ventricular assist devices (LVAD) are increasingly being used as a therapeutic option for advanced heart failure, both as a bridge to heart transplant (HT) and, given the rapid advances in the LVAD’s functionality and durability, and the limited availability of donor organs, as long-term destination therapy (Kirklin et al., 2017). The implant of a LVAD comes with a 48% reduction in mortality from any cause (Rose et al., 2009) and an improvement in quality of life (Rogers et al., 2010), although the patient often faces difficulties transitioning into their new life, and can suffer from a number of adverse events, like bleedings and infections (Adams and Wrightson, 2018; Han et al., 2018).

There is an increase in the number of LVAD candidates, caused by individuals with advanced and end-stage heart failure who do not have access to (or are not eligible for) HT. Consequently, there is an increasing number of LVAD recipients (more than 3,000 new implants in 2019 alone, according to the Society of Thoracic Surgeons’ 2020 annual report; Molina et al., 2021), who are living longer with the implant. Following these perspectives, it is crucial to assess the patients to determine their level of functional capacity, in order to improve the patient’s physical conditioning. In this optic, while aerobic-cardiovascular variables (e.g., power, capacity, etc.) is abundantly monitored and assessed in those patients, muscular strength-related conditioning is less well expressed in scientific literature.

Noteworthy, handgrip strength has been found to be inversely correlated with hospital length of stay after LVAD implantation (Yost and Bhat, 2017), and sarcopenia diagnosed before the implantation is associated with a decreased 6-months survival ratio (Roehrich et al., 2022). To our knowledge, the only study investigating the lower limbs’ strength performance of LVAD recipients was conducted by Kerrigan et al. (2013), who reported data only about isokinetic test of knee extension, concluding that peak torque was strongly associated with patient-reported health status. Previous research has found significant correlation between handgrip strength and VO_2_ peak both in younger (Dag et al., 2021; Ajepe et al., 2022) and older people (Sugie et al., 2018); however, no such relationship is apparent between leg strength and VO_2_ peak, at least in older, inactive individuals (Matthews et al., 2020).

On one hand, strength quantification is fundamental in predicting the independence of daily living activities in LVAD patients, in fact muscular strength has also been found to be a determinant of physical disability (Savage et al., 2011); on the other hand, understanding muscular strength in LVAD recipients is relevant to increase the overall fitness through structured training protocols, in fact resistance training can provide an increase in muscle strength, aerobic power and capacity and quality of life in this population (Giuliano et al., 2017), compared with usual care (Ganga et al., 2017). However, the programs used are highly variable, as no official guidelines have been published at this point (Alonso et al., 2021). In the light of these viewpoints and in the lack of a solid body of knowledge, this study aims to analyze strength parameters in subjects with LVAD, and the potential correlation between muscular strength and aerobic capacity in LVAD patients.

## Materials and methods

Between 2015 and 2019, 38 LVAD recipients (33 men and five women, age: 58.1 ± 7.64 years) received a medical examination. All participants gave their informed consent, and the study was approved by the local ethics committee (Padova University).

Participants’ height and weight (body mass) were measured respectively with a stadiometer (Ayrton Corporation, Model S100, Prior Lake, MN, United States), and an electronic scale (Home Health Care Digital Scale, Model GS 51 XXL, Beuer Gmbh, Ulm, Germany). Height and weight (body mass) were used to calculate body mass index (BMI) of the participants. The medical history, medical examination, and cardiopulmonary exercise test were administered by a physician with Sport Medicine specialization. Exercise capacity was assessed by incremental, ECG-monitored, cardiopulmonary exercise testing (Jaeger- Masterscreen-CPX, Carefusion, Germany). Both tests were randomly performed on treadmill (modified Bruce protocol) and bicycle (protocol +10 W/min), and performed until exhaustion (Borg rating of perceived exertion (RPE) ≥18/20).

The New York Heart Association (NYHA) classification was applied to classified patients in one of four categories based on their limitations during physical activity. The limitations/symptoms are in regards to normal breathing and varying degrees in shortness of breath and or angina pain (Heart Foundation, 2014). Charlson comorbidity index (Charlson et al., 1987) were used to classify the patient health status in relation to comorbid conditions.

Before muscular strength tests, a warm-up was performed to reduce the risk of injuries. A 60-s recovery period was allowed between all testing procedures. Dominant and non-dominant handgrip strength was measured with a calibrated dynamometer (Baseline, Elmsford, NY, United States). Grip handle was adjusted to accommodate the size and comfort of the participant’s hand, and the elbow was flexed to 90° to guarantee the strongest grip strength measurement (Mathiowetz et al., 1985). Three trials for each hand were performed, and the mean of dominant hand was used for percentile identification. Lower limb muscle strength tests were performed with subjects seated on the multi-joint system with the backrest angled at 90° to the seat. Belts were fastened across the thighs, pelvis, and shoulders to minimize body movements and to optimally isolate the movement of the knee and ankle joints. Subjects folded their arms across their chest and were not permitted to hold on to the equipment during the tests. During knee trials, the lever fulcrum was aligned with the rotation axis of knee, with the lateral femoral epicondyle used as the point of reference, and the shin pad was placed 2 cm above the medial malleoli. Instead, during the ankle trials, the lever fulcrum was aligned with the medial malleoli. Before all isokinetic tests, the weight of the legs and the ankles were noted and a gravity adjustment was made using the computer software. During the maximal isometric knee extension, the lever arm was set at 75° extension, calculated from the maximum knee extension of each participant. Subjects had to push as much as possible, with leg, on the shin pad for 5 s. Conversely, during maximal isokinetic knee extension and flexion participants pushed and pulled the shin pad as fast as possible for five times uninterrupted. The velocity of isokinetic movement was set at 90°s. When testing the maximal isometric ankle plantar and dorsal flexion, the lever arm was set at 30° of plantar flexion, calculated from the maximum ankle dorsal flexion (0°) of each participant, and the foot was fixed on a support with two stripes. Subjects had to push down and pull up the ankle support as much as possible for 5 s, during extension and flexion trials. Finally, during maximal isokinetic ankle plantar and dorsal flexion, participants had to push down and pull up the ankle support as fast as possible for five times continuously. The velocity of this isokinetic movement was set at 90°s. All data were acquired at 1,000 Hz, and analyzed as absolute strength, and relative strength (absolute strength/body mass). This protocol was previously used and validated for older adults (Bergamin et al., 2017).

Postural control was measured by means of posturography with an ARGO stabilometric platform (RGMD, Genova, IT) in three conditions: Eyes open, eyes closed and dual task (counting backwards aloud), as previously outlined in Zanotto et al. (2020). Each test was performed with subject upright with feet together and the arms at sides. In front of it, a blackboard was placed to the distance of 3 m. During the Romberg test with eyes open, the subject has to fixed a reference point located on the blackboard for 30 s. During the Romberg test with eyes closed, the subject has to stay on the platform for 30 s with closed eyes. In dual-task condition, participants had to stand as still as possible in Romberg position, with eyes open, counting backwards aloud, starting from a randomly selected number, in steps of one, as fast and as accurately as possible for the entire duration of the test (Yardley et al., 1999; Bergamin et al., 2014). All participants performed randomly the three balance tests three times.

Given the study design, a descriptive statistical approach was chosen (data is presented as mean ± S.D.), and the Pearson product moment correlation coefficients between VO_2_ peak and strength measurements were computed (*α* = 0.05). All data was managed using Microsoft Excel 365 (Microsoft Corporation, 2018).

## Results

38 participants underwent functional capacity, strength, and balance assessments, as reported in the method section. Results from tests are presented in Table 1, and data are expressed as mean ± standard deviation. Mean BMI was 27.26 ± 3.97, with 12 participants classified as “Obese” (BMI>29.9). The physical and clinical evaluations were conducted after a mean period of 59.19 ± 46.37 weeks. Data on pharmacological therapies revealed the participants were taking, on average, 10 different medications each day. In addition to the medications taken for the management of heart failure, pain and infection risk, it should be noted that 47% of the participants were taking at least one psychotropic medication (antidepressants being the most common).

**TABLE 1:** Test Results.

Outcome	*n*	Mean ± S.D.	Outcome	*n*	Mean ± S.D.
VO_2_ Peak (L/min kg)	18	12.38 ± 3.43	Isokinetic extension strength, left ankle (kg)	24	12.81 ± 7.18
Handgrip, dominant hand (kg)	38	30.05 ± 10.61	Isokinetic flexion strength, left ankle (kg)	24	16.29 ± 5.72
Handgrip, non dominant hand (kg)	37	26.59 ± 9.73	Romberg test, eyes open, sway path (mm/s)	31	19.44 ± 6.36
Isometric extension strength, right knee (kg)	28	120.86 ± 44.52	Romberg test, eyes open, sway area (mm^2^/s)	31	46.47 ± 22.98
Isometric extension strength, left knee (kg)	28	109.57 ± 38.95	Romberg test, eyes open, anterior-posterior oscillations (mm)	31	25.68 ± 8.03
Isokinetic extension strength, right knee (kg)	27	80.68 ± 28.84	Romberg test, eyes open, lateral oscillations (mm)	31	31.95 ± 7.62
Isokinetic flexion strength, right knee (kg)	27	35.47 ± 17.96	Romberg test, eyes closed, sway path (mm/s)	31	30.65 ± 12.86
Isokinetic extension strength, left knee (kg)	27	73.14 ± 21.71	Romberg test, eyes closed, anterior-posterior oscillations (mm)	31	35.32 ± 11.55
Isokinetic flexion strength, left knee (kg)	27	37.02 ± 18.94	Romberg test, dual task, sway path (mm/s)	29	23.02 ± 8.19
Isometric extension strength, right ankle (kg)	24	19.38 ± 10.53	Romberg test, dual task, anterior-posterior oscillations (mm)	29	26.63 ± 9.67
Isometric flexion strength, right ankle (kg)	24	28.51 ± 7.31	Romberg test, eyes closed, sway area (mm^2^/s)	31	94.88 ± 63.60
Isometric extension strength, left ankle (kg)	23	17.58 ± 12.30	Romberg test, eyes closed, lateral oscillations (mm)	31	41.77 ± 12.90
Isometric flexion strength, left ankle (kg)	23	26.88 ± 6.51	Romberg test, dual task, sway area (mm^2^/s)	29	53.24 ± 28.68
Isokinetic extension strength, right ankle (kg)	25	11.83 ± 6.51	Romberg test, dual task, lateral oscillations (mm)	29	31.80 ± 8.79
Isokinetic flexion strength, right ankle (kg)	25	16.48 ± 6.72			

The most common comorbidity was type II diabetes (7 participants), followed by chronic kidney disease, dyslipidemia and hypertension (5 participants), with a mean Charlson Comorbidity Index (computable for 23 participants) of 3.48 ± 1.41 resulting in a 60% 10-years survival rate (drug therapies and comorbidities are described in Table 2). Participants tested showed a poor overall physical condition and a weak functional capacity, with most of the participants (12 out of 18 participants) who completed the CPET examination falling into the “C” (10 participants) or “D” (2 participants) functional class of the NYHA classification.

**TABLE 2:** Characteristics of Study Participants.

Outcome	Result (n of participants)
Age (years)	58.11 ± 7.64 (36)
Stature (m)	1.70 ± 0.07 (36)
Body mass (kg)	79.26 ± 14.54 (36)
BMI (kg/m^2^)	27.26 ± 3.97 (36)
Comorbidities (type)	Type II Diabetes Mellitus (7), hypertension (5), dyslipidemia (5), chronic kidney disease (5), hernia (inguinal, umbilical, hiatal) (3), gastritis (2), Gallbladder calculosis or cholecystectomy (2), other (10)
Comorbidities (*n*. of)	No com. (5), 1 com. (8), 2 com. (4), 3 com. (4), 4 com. (3), 5 com. (1), ≥6 com. (0)
Charlson Comorbidity Index	3.48 ± 1.41 (23)
Drugs (type)	anticoagulant (18), diuretics (18), proton pump inhibitors (18), Antiarrhythmic (13), non-steroidal antinflammatory (12), mineral supplements (Calcium, sodium, magnesium, potassium) (12), iron supplement (13), antihypertensive (10), antithrombotic (9), antibiotic (9), beta-blocker (8), thyroid hormonal drugs (8), statin (8), antidepressive (Other) (5), diabetes medication (4), gout medication (4), benzodiazepine (4), vitamin supplements (B, D) (4), hepatic medication (3), alpha-blocker (3), antipsychotic (3), antidepressive (SSRI) (2), other (10)
Drugs (n. of)	4 drugs (1), 7 drugs (3), 8 drugs (4), 9 drugs (3), 10 drugs (2), 11 drugs (5), 14 drugs (2), 15 drugs (1), 16 drugs (1)

Seven participants had a HeartWare HVAD System implanted, which was recalled in 2021 after a series of malfunctions (Medical Device Recalls, 2021); fortunately, no pump-related adverse events were registered during or between the tests, and no systematic difference in performance could be observed between these and other participants.

Only 12 participants (32%) were able to successfully complete all the assessments. CPET and isokinetic strength trials were the least tolerated tests (completed by 18 and 22 participants, respectively). Handgrip test has been completed bilaterally by 37 participants. Mean VO_2_ peak was 12.38 ± 3.43 ml/kg/min, and the mean handgrip strength (dominant hand) was 30.05 ± 10.61 Kg. The balance tests were completed by 31 participants in the eyes open/closed condition, and by 29 participants in the dual task condition; in the eyes open condition mean sway path was 19.44 ± 6.36 mm/s, mean sway area was 46.47 ± 22.98 mm^2^/s, mean anterior-posterior oscillations were 25.68 ± 8.03 mm, and mean lateral oscillations were 31.95 ± 7.62 mm. In the eyes closed condition mean sway path was 30.65 ± 12.86 mm/s, mean sway area was 94.88 ± 63.60 mm^2^/s, mean anterior-posterior oscillations were 35.32 ± 11.55 mm, and mean lateral oscillations were 41.77 ± 12.90 mm. In the dual task condition, mean sway area was 23.02 ± 8.19 mm/s, mean sway area was 53.24 ± 28.68 mm^2^/s, mean anterior-posterior oscillations were 26.63 ± 9.67 mm, and mean lateral oscillations were 31.80 ± 8.79 mm.

No statistically significant correlations were found between VO_2_ peak and handgrip strength (*p* > 0.2) and between VO_2_ with any of the lower limb strength measures (*p* > 0.1), as outlined in Table 3.

**TABLE 3:** Correlation.

Outcome	*n*	VO_2_ peak correlation (Pearson’s r)	*p* value
Handgrip, dominant hand (kg)	18	−0.31	0.21
Handgrip, non dominant hand (kg)	18	−0.1	0.71
Isometric extension strength, right knee (kg)	14	−0.38	0.18
Isometric extension strength, left knee (kg)	14	−0.41	0.14
Isokinetic extension strength, right knee (kg)	14	−0.3	0.30
Isokinetic flexion strength, right knee (kg)	14	−0.28	0.34
Isokinetic extension strength, left knee (kg)	14	−0.17	0.57
Isokinetic flexion strength, left knee (kg)	14	−0.27	0.34
Isometric extension strength, right ankle (kg)	14	0.26	0.38
Isometric flexion strength, right ankle (kg)	14	−0.25	0.38
Isometric extension strength, left ankle (kg)	13	0.45	0.12
Isometric flexion strength, left ankle (kg)	13	−0.04	0.91
Isokinetic extension strength, right ankle (kg)	14	−0.02	0.95
Isokinetic flexion strength, right ankle (kg)	14	−0.03	0.93
Isokinetic extension strength, left ankle (kg)	13	−0.02	0.96
Isokinetic flexion strength, left ankle (kg)	13	−0.24	0.43

n: number of participants who completed both the VO2 peak and corresponding strength assessment.

## Discussion

The aim of this paper was to characterize some aspects of physical capacity in patients with LVAD. In particular, the strength profile was analyzed using isometric (including the handgrip test) and isokinetic strength tests. Compared to a normal population in terms of age, body mass and gender; in general, muscle strength of LVAD patients appeared as reduced. With more detail, 25 participants were below the 25° percentile of their population’s normative reference values for handgrip strength, furthermore 10 of which were below the 5° percentile. Mean isometric knee extension strength was below the reference values found in literature for a comparable (healthy) population; the reference values used are outlined in Šarabon, Kozinc, and Perman (2021), who employed a similar methodology for determining isometric knee strength. Reference values from a healthy population were chosen because, to our knowledge, there are no reference values for LVAD recipients strength parameters. This drop in both upper and lower-body strength could be explained by a few possible reasons. First off, although more active than patients with heart failure, LVAD recipients demonstrated lower levels of physical activity than healthy subjects (Jakovljevic et al., 2014). This condition, in turn, could explain a reduction in muscle mass and functionality, especially in older adults (Rezuş et al., 2020). Secondly, LVAD recipients (and before, heart failure patients) often experienced prolonged and repeated periods of bed rest, which can lead to muscle atrophy (Ikezoe et al., 2011) with potential consequential impairment in terms of muscle strength. Finally, subjects with reduced heart functionality often suffered from skeletal muscle dysfunction, contributing to exercise intolerance (Haykowsky et al., 2011; Bekfani et al., 2020). This vicious circle could resemble positive feedback where, however, muscular strength could progressively decrease over time.

Literature was clear about how upper and lower limb strength contribute to older adults’ ability to complete activities of daily living (Wang et al., 2020); knee and ankle extension strength can be used as predictors of loss of autonomy (Buckinx et al., 2019), therefore understanding the decline in strength measure become important for LVAD patients to counteract the risk for disability. Additionally, establishing robust strength training protocols to maintain sufficient muscle function assumes a critical role for these patients.

Analyzing the aerobic-cardiovascular component, as could be expected, aerobic capacity was also compromised in this population: out of the 18 participants who completed the CPET, 15 were below 50% of their predicted maximal VO_2_, six of which were below 30% VO_2_ max. VO_2_ has been previously found to be a predictor of long-term survival in the general population (Ross et al., 2016), and in heart-failure patients (Hsich et al., 2007).

While the association between muscle mass and aerobic capacity waned with aging (Kim et al., 2016), muscle loss appeared to play a pivotal role in age-related decline of VO_2_ max (Fleg and Lakatta, 1988). In trained older men and women, instead, the reduction in max O_2_ delivery seemed to be the main driving factor of this phenomenon (Proctor and Joyner, 1997), and lower-limb strength was positively correlated with maximal aerobic performance in trained older individuals (Matthews et al., 2020). In LVAD recipients, instead, muscle strength (as measured by isometric and isokinetic tests) was not significantly correlated with VO_2_ peak. This could signify that aerobic capacity of those patients was probably so compromised that cardiac performance remained the limiting factor during CPET, which highlighted the importance of performing specific strength tests to accurately describe the functional status of this population.

Another important issue concerned balance test is that all parameters (sway path, sway area, anterior-posterior and medio-lateral oscillations) were greater in closed eyes condition respect to open eyes condition or dual-task condition. This finding suggests that LVAD recipients seem to have a compromised postural control with visual deprivation. This probably due to physical decondition and in some cases for the side effects of certain drug therapies (e.g. antiarrhythmic) or other comorbidities (e.g. diabetes) that may alter proprioception. One aspect to consider is the presence of the LVAD’s external battery pack, which may have altered the test. Indeed, this can weigh up to 2 kg with probable alteration in the subject’s sway parameters. For these reasons to assess the risk of falls, a graded test, like the Berg balance scale (Berg et al., 1992) could be more appropriate in this population. Alternatively, the risk of falls could be inferred from the knee and ankle peak torque and rate of force development (Bento et al., 2010; Valenzuela et al., 2020), even though more population-specific trials would be needed to confirm this assumption.

The overrepresentation of male participants in this study (33/38 participants) is in line with other reports, where women were less likely to receive a LVAD, since they appear to have poorer outcomes and more frequent adverse events (Joyce et al., 2009; Magnussen et al., 2018; Dayanand et al., 2021). The high prevalence of obesity (over 30% of participants) in the sample should spark action towards weight management strategies in this population. Obesity is a known general risk factor for cardiovascular diseases, and has been found to increase the incidence of infections, neurological complications, and thrombosis in LVAD recipients, affecting short-term survival (Zhigalov et al., 2020). The high percentage of participants under a psychotropic medication therapy is compatible with the psychological challenges faced by these persons in the transitional period following the LVAD implant (Okam et al., 2020).

This study presented several limitations. The first issue is obviously linked to the great difficulty in finding this type of patient and especially in testing them. Secondarily, there was a considerable amount of data loss, mainly related to the health status of the participants. These missing data were not random but came mostly from the participants who could not complete the physical tests (or for whom the tests had to be ended prematurely), which means that these results were computed using only the data from the fittest participants, and thus the means are likely to be overestimated, especially for the outcomes with the lowest sample sizes. Finally, during the recruiting process, we did not impose inclusion/exclusion criteria on the time period between LVAD implant and examination, unavoidably reducing the homogeneity of the sample. This variability is probably a feature of the population itself, which nevertheless makes the interpretation of the data more complex, even compared with the simple normal population in terms of comparison.

In conclusion, LVAD recipients show greatly reduced functional capacity and tolerance to exercise and exercise testing, with low overall strength levels. This complex frame therefore entails a reduced physical function. As strength performance appears to be independent from VO2 peak, strength tests, such as the handgrip strength test, should be included in the physical function assessment; however, given that isokinetic tests, especially of the ankle joint muscles, were poorly tolerated, a different alternative should be explored in this population. Future works on LVAD recipients could focus on comparing different physical testing protocols to find the most suitable compromise between validity and applicability for these persons. Further studies could help establishing reference values for strength and cardiovascular outcomes that are specific to LVAD recipients, to use as starting point when preparing an exercise training protocol and exercise guidelines for LVAD recipients.

## Data Availability

The original contributions presented in the study are included in the article/Supplementary Material, further inquiries can be directed to the corresponding author.

## References

[B1] Adams E. E. Wrightson M. L. (2018). Quality of life with an LVAD : A misunderstood concept. Heart & Lung 47 (3), 177–183. 10.1016/j.hrtlng.2018.02.003 29551363

[B2] Ajepe O. Mgbemena N. Okafor U. Ehuwa O. Okeke C. Osundiya O. (2022). Relationship between estimated VO2max and handgrip strength in healthy young Nigerian adults. Internet J. Allied Health Sci. Pract. 20 (1), 17. 10.46743/1540-580x/2022.2079

[B3] Alonso W. W. Ryan T. R. Lundgren S. W. Tlusty G. Castleberry A. W. Pozehl B. J. (2021). Clinicians call for post left ventricular assist device implantation physical activity guidelines. ASAIO J. Am. Soc. Artif. Intern. Organs 67 (10), e166–e168. 10.1097/MAT.0000000000001343 33528166

[B4] Bekfani T. Bekhite Elsaied M. Derlien S. Nisser J. Westermann M. Nietzsche S. (2020). Skeletal muscle function, structure, and metabolism in patients with heart failure with reduced ejection fraction and heart failure with preserved ejection fraction. Circ. Heart Fail. 13, e0007198. 10.1161/CIRCHEARTFAILURE.120.007198 33302709

[B5] Bento P. C. B. Pereira G. Ugrinowitsch C. Rodacki A. L. F. (2010). Peak torque and rate of torque development in elderly with and without fall history. Clin. Biomech. 25 (5), 450–454. 10.1016/J.CLINBIOMECH.2010.02.002 20350773

[B6] Berg K. O. Wood-Dauphinee S. L. Williams J. I. Maki B. (1992). Measuring balance in the elderly: Validation of an instrument. Can. J. Public Health = Revue Can. de Sante Publique 83 (2), S7–S11. 1468055

[B7] Bergamin M. Gobbo S. Bullo V. Vendramin B. Duregon F. Frizziero A. (2017). Reliability of a device for the knee and ankle isometric and isokinetic strength testing in older adults. Muscles Ligaments Tendons J. 7 (2), 323–330. 10.11138/MLTJ/2017.7.2.323 29264344 PMC5725182

[B8] Bergamin M. Gobbo S. Zanotto T. Sieverdes J. C. Alberton C. L. Zaccaria M. (2014). Influence of age on postural sway during different dual-task conditions. Front. Aging Neurosci. 6, 271. 10.3389/fnagi.2014.00271 25374539 PMC4205805

[B9] Buckinx F. Croisier J. L. Charles A. Petermans J. Reginster J. Y. Rygaert X. (2019). Normative data for isometric strength of 8 different muscle groups and their usefulness as a predictor of loss of autonomy among physically active nursing home residents: The senior cohort. J. Musculoskelet. Neuronal Interact. 19 (3), 258–265. 31475932 PMC6737556

[B10] Charlson M. E. Pompei P. Ales K. L. MacKenzie C. R. (1987). A new method of classifying prognostic comorbidity in longitudinal studies: Development and validation. J. Chronic Dis. 40 (5), 373–383. 10.1016/0021-9681(87)90171-8 3558716

[B11] Dag F. Tas S. Cimen O. B. (2021). Hand-grip strength is correlated with aerobic capacity in healthy sedentary young females. Monten. J. Sports Sci. Med. 10 (1), 55–60. 10.26773/mjssm.210308

[B12] Dayanand S. Martinez J. M. Figueredo V. M. Gupta S. (2021). Mechanical circulatory support in women. J. Cardiol. 77 (3), 209–216. 10.1016/J.JJCC.2020.07.012 32868140

[B13] Fleg J. L. Lakatta E. G. (1988). Role of muscle loss in the age-associated reduction in $\dot{\text{V}}$ (O2 max). J. Appl. Physiol. 65 (3), 1147–1151. 10.1152/jappl.1988.65.3.1147 3182484

[B14] Ganga H. V. Leung A. Jantz J. Choudhary G. Stabile L. Levine D. J. (2017). Supervised exercise training versus usual care in ambulatory patients with left ventricular assist devices: A systematic review. PLOS ONE 12 (3), e0174323. 10.1371/JOURNAL.PONE.0174323 28362876 PMC5375157

[B15] Giuliano C. Karahalios A. Neil C. Allen J. Levinger I. (2017). The effects of resistance training on muscle strength, quality of life and aerobic capacity in patients with chronic heart failure — a meta-analysis. Int. J. Cardiol. 227, 413–423. 10.1016/j.ijcard.2016.11.023 27843045

[B16] Han J. J. Acker M. A. Atluri P. (2018). Left ventricular assist devices. Circulation 138 (24), 2841–2851. 10.1161/CIRCULATIONAHA.118.035566 30565993

[B17] Haykowsky M. J. Brubaker P. H. John J. M. Stewart K. P. Morgan T. M. Kitzman D. W. (2011). Determinants of exercise intolerance in elderly heart failure patients with preserved ejection fraction. J. Am. Coll. Cardiol. 58 (3), 265–274. 10.1016/j.jacc.2011.02.055 21737017 PMC3272542

[B18] Heart Foundation. (2014). New York heart association (NYHA) classification. October 2011, 1.

[B19] Hsich E. Chadalavada S. Krishnaswamy G. Starling R. C. Pothier C. E. Blackstone E. H. (2007). Long-term prognostic value of peak oxygen consumption in women versus men with heart failure and severely impaired left ventricular systolic function. Am. J. Cardiol. 100 (2), 291–295. 10.1016/j.amjcard.2007.02.096 17631085

[B20] Ikezoe T. Mori N. Nakamura M. Ichihashi N. (2011). Effects of age and inactivity due to prolonged bed rest on atrophy of trunk muscles. Eur. J. Appl. Physiol. 112 (1), 43–48. 10.1007/S00421-011-1952-X 21472438

[B21] Jakovljevic D. G. McDiarmid A. Hallsworth K. Seferovic P. M. Ninkovic V. M. Parry G. (2014). Effect of left ventricular assist device implantation and heart transplantation on habitual physical activity and quality of life. Am. J. Cardiol. 114 (1), 88–93. 10.1016/J.AMJCARD.2014.04.008 24925802 PMC4061472

[B22] Joyce D. L. Conte J. V. Russell S. D. Joyce L. D. Chang D. C. (2009). Disparities in access to left ventricular assist device therapy. J. Surg. Res. 152 (1), 111–117. 10.1016/J.JSS.2008.02.065 18466923

[B23] Kerrigan D. J. Williams C. T. Ehrman J. K. Bronsteen K. Saval M. A. Schairer J. R. (2013). Muscular strength and cardiorespiratory fitness are associated with health status in patients with recently implanted continuous-flow LVADs. J. Cardiopulm. Rehabil. Prev. 33 (6), 396–400. 10.1097/HCR.0000000000000024 24189213

[B24] Kim C. H. Wheatley C. M. Behnia M. Johnson B. D. (2016). The effect of aging on relationships between lean body mass and VO2max in rowers. PLoS ONE 11 (8), e0160275. 10.1371/journal.pone.0160275 27479009 PMC4968829

[B25] Kirklin J. K. Pagani F. D. Kormos R. L. Stevenson L. W. Blume E. D. Myers S. L. (2017). Eighth annual INTERMACS report: Special focus on framing the impact of adverse events. J. Heart Lung Transplant. 36 (10), 1080–1086. 10.1016/J.HEALUN.2017.07.005 28942782

[B26] Lippi G. Sanchis-Gomar F. (2020). Global epidemiology and future trends of heart failure. AME Med. J. 5 (0), 15. 10.21037/AMJ.2020.03.03

[B27] Magnussen C. Bernhardt A. M. Ojeda F. M. Wagner F. M. Gummert J. de By T. M. M. H. (2018). Gender differences and outcomes in left ventricular assist device support: The European Registry for Patients with Mechanical Circulatory Support. J. Heart Lung Transpl. 37 (1), 61–70. 10.1016/J.HEALUN.2017.06.016 28754423

[B28] Mathiowetz V. Rennells C. Donahoe L. (1985). Effect of elbow position on grip and key pinch strength. J. Hand Surg. Am. 10 (5), 694–697. 10.1016/S0363-5023(85)80210-0 4045150

[B29] Matthews H. Warren V. Graff R. Markofski M. (2020). "Positive relationship between VO2max and leg strength in healthy older adults who regularly exercise, but not in those who do not exercise," in International journal of exercise science: conference proceedings 2 (12). Available at: https://digitalcommons.wku.edu/ijesab/vol2/iss12/164 .

[B30] Medical Device Recalls (2021). “Medtronic stops distribution and sale of HeartWare HVAD system due to risl of neurological adverse events, mortality, and potential failure to restart,” in Medical device recalls. Available at: https://www.fda.gov/medical-devices/medical-device-recalls/medtronic-stops-distribution-and-sale-heartware-hvad-system-due-risk-neurological-adverse-events .

[B47] Molina E. J. Shah P. Kiernan M. S. Cornwell W. K. Copeland H. Takeda K. (2021). The Society of Thoracic Surgeons Intermacs 2020 Annual Report.. Ann. Thorac. Surg. 111 (3), 778–792. 10.1016/j.athoracsur.2020.12.038 33465365

[B31] Okam N. A. Ahmad W. Rana D. Torrilus C. Jahan N. Sedrakyan S. (2020). Psychological spectrum experienced by heart failure patients after left ventricular assist device implantation. Cureus 12 (8), e9671. 10.7759/CUREUS.9671 32923266 PMC7485994

[B32] Proctor D. N. Joyner M. J. (1997). Skeletal muscle mass and the reduction of VO2max in trained older subjects. J. Appl. Physiol. (1985) 82 (5), 1411–1415. 10.1152/JAPPL.1997.82.5.1411 9134886

[B33] Rezuş E. Burlui A. Cardoneanu A. Rezuş C. Codreanu C. Pârvu M. (2020). Inactivity and skeletal muscle metabolism: A vicious cycle in old age. Int. J. Mol. Sci. 21 (2), 592. 10.3390/IJMS21020592 PMC7014434 31963330

[B34] Roehrich L. Sündermann S. H. Just I. A. Kopp Fernandes L. Stein J. Solowjowa N. (2022). Comparison of feasibility and results of frailty assessment methods prior to left ventricular assist device implantation. Esc. Heart Fail. 9 (2), 1038–1049. 10.1002/EHF2.13764 34994094 PMC8934953

[B35] Rogers J. G. Aaronson K. D. Boyle A. J. Russell S. D. Milano C. A. Pagani F. D. (2010). Continuous flow left ventricular assist device improves functional capacity and quality of life of advanced heart failure patients. J. Am. Coll. Cardiol. 55 (17), 1826–1834. 10.1016/J.JACC.2009.12.052 20413033

[B36] Rose E. A. Gelijns A. C. Moskowitz A. J. Heitjan D. F. Stevenson L. W. Dembitsky W. (2009). Long-term use of a left ventricular assist device for end-stage heart failure. N. Engl. J. Med. 345 (20), 1435–1443. 10.1056/NEJMoa01217510.1056/NEJMOA012175 11794191

[B37] Ross R. Blair S. N. Arena R. Church T. S. Després J. P. Franklin B. A. (2016). Importance of assessing cardiorespiratory fitness in clinical practice: A case for fitness as a clinical vital sign: A scientific statement from the American heart association. Circulation 134 (24), e653–e699. 10.1161/CIR.0000000000000461 27881567

[B38] Šarabon N. Kozinc Ž. Perman M. (2021). Establishing reference values for isometric knee extension and flexion strength. Front. Physiol. 12, 1809. 10.3389/fphys.2021.767941 PMC8554160 34721087

[B39] Savage P. A. Shaw A. O. Miller M. S. Vanburen P. Lewinter M. M. Ades P. A. (2011). Effect of resistance training on physical disability in chronic heart failure. Med. Sci. Sports Exerc. 43 (8), 1379–1386. 10.1249/MSS.0b013e31820eeea1 21233772 PMC3410739

[B40] Sugie M. Harada K. Takahashi T. Nara M. Ishikawa J. Tanaka J. (2018). Relationship between hand grip strength and peak VO2 in community-dwelling elderly outpatients. JCSM Clin. Rep. 3 (1). 10.17987/jcsm-cr.v3i1.48

[B41] Valenzuela P. L. Maffiuletti N. A. Saner H. Schütz N. Rudin B. Nef T. (2020). Isometric strength measures are superior to the timed up and go test for fall prediction in older adults: Results from a prospective cohort study. Clin. Interv. Aging 15, 2001–2008. 10.2147/CIA.S276828 33149561 PMC7602904

[B42] Wang D. X. M. Yao J. Zirek Y. Reijnierse E. M. Maier A. B. (2020). Muscle mass, strength, and physical performance predicting activities of daily living: A meta-analysis. J. Cachexia, Sarcopenia Muscle 11 (1), 3–25. 10.1002/jcsm.12502 31788969 PMC7015244

[B43] Yardley L. Gardner M. Leadbetter A. Lavie N. (1999). Effect of articulatory and mental tasks on postural control. NeuroReport 10 (2), 215–219. 10.1097/00001756-199902050-00003 10203311

[B44] Yost G. Bhat G. (2017). Relationship between handgrip strength and length of stay for left ventricular assist device implantation. Nutr. Clin. Pract. 32 (1), 98–102. Official Publication of the American Society for Parenteral and Enteral Nutrition. 10.1177/0884533616665926 27600398

[B45] Zanotto T. Gobbo S. Bullo V. Vendramin B. Roma E. Duregon F. (2020). Postural balance, muscle strength, and history of falls in end-stage renal disease patients living with a kidney transplant: A cross-sectional study. Gait Posture 76, 358–363. 10.1016/J.GAITPOST.2019.12.031 31901763

[B46] Zhigalov K. Oliveira Sá M. P. B. Rad A. A. Vardanyan R. Goerdt L. Chrosch T. (2020). The impact of obesity on left ventricular assist device outcomes. Medicina 56 (11), E556. 10.3390/MEDICINA56110556 33113962 PMC7690722

